# Structural evolution drives diversification of the large LRR‐RLK gene family

**DOI:** 10.1111/nph.16455

**Published:** 2020-02-29

**Authors:** Jarrett Man, Joseph P. Gallagher, Madelaine Bartlett

**Affiliations:** ^1^ Biology Department University of Massachusetts Amherst 611 North Pleasant Street, 221 Morrill 3 Amherst MA 01003 USA

**Keywords:** domain loss, gene trees, leucine‐rich repeat, LRR‐RLK evolution, molecular phylogenetics, protein evolution, receptor‐like kinase

## Abstract

● Cells are continuously exposed to chemical signals that they must discriminate between and respond to appropriately. In embryophytes, the leucine‐rich repeat receptor‐like kinases (LRR‐RLKs) are signal receptors critical in development and defense. LRR‐RLKs have diversified to hundreds of genes in many plant genomes. Although intensively studied, a well‐resolved LRR‐RLK gene tree has remained elusive.
● To resolve the LRR‐RLK gene tree, we developed an improved gene discovery method based on iterative hidden Markov model searching and phylogenetic inference. We used this method to infer complete gene trees for each of the LRR‐RLK subclades and reconstructed the deepest nodes of the full gene family.
● We discovered that the LRR‐RLK gene family is even larger than previously thought, and that protein domain gains and losses are prevalent. These structural modifications, some of which likely predate embryophyte diversification, led to misclassification of some LRR‐RLK variants as members of other gene families. Our work corrects this misclassification.
● Our results reveal ongoing structural evolution generating novel LRR‐RLK genes. These new genes are raw material for the diversification of signaling in development and defense. Our methods also enable phylogenetic reconstruction in any large gene family.

## Introduction

Developmental and defense processes are cued by complex mixtures of extracellular chemical signals. Cells produce receptor proteins to detect these signals and, in turn, to direct downstream cellular responses. Just as there are many signals, there are many receptors for these signals, and some receptors are in large gene families. The leucine‐rich repeat receptor‐like kinase (LRR‐RLK) receptors, in particular, comprise the largest plant‐specific clade of the eukaryotic kinase superfamily (Shiu & Bleecker, 2001a). To perceive and relay extracellular signals, most LRR‐RLKs localize to the plasma membrane, where each LRR‐RLK has an extracellular LRR domain, a single‐pass transmembrane domain, and a cytosolic RLK domain. LRR‐RLKs remain inactive until a signal ligand is bound by the extracellular LRR domain, upon which LRR‐RLKs oligomerize to form active complexes (Diévart & Clark, 2004; Meng *et al.*, 2016; Santiago *et al.*, 2016). Once activated, the cytosolic RLK domain can trigger an intracellular signaling cascade to modify cellular activity (He *et al.*, 2018). LRR domains can have exquisite signal specificity and sensitivity, and RLK domains can selectively phosphorylate many proteins downstream of signal perception (Shiu & Bleecker, 2003; Santiago *et al.*, 2016; He *et al.*, 2018; Je *et al.*, 2018).

This versatile system of signal perception and transduction has expanded to a large family of hundreds of genes per genome (Shiu & Bleecker, 2003). LRR‐RLKs control many plant developmental processes, such as stomatal patterning, vasculature organization, branching architecture, and pollen tube guidance (Bommert *et al.*, 2005; Fisher & Turner, 2007; Qian *et al.*, 2018; Johnson *et al.*, 2019). They are also used extensively in defense against pathogens (Diévart & Clark, 2004; Huffaker & Ryan, 2007; Sakamoto *et al.*, 2012; Peng & Kaloshian, 2014). Some impact agronomically important developmental processes and can impact yield, making them appealing targets for crop improvement (Diévart & Clark, 2004; Song *et al.*, 2015; Je *et al.*, 2016; Rodríguez‐Leal *et al.*, 2017; Lemmon *et al.*, 2018).

Despite the importance of this family to plant development, defense, and agriculture, phylogenetic characterization remains incomplete, and this impedes research. For example, high rates of functional redundancy in this family often obscure function in single gene mutants (Nowak *et al.*, 1997; Sieburth, 2007; Nimchuk *et al.*, 2015; Rodriguez‐Leal *et al.*, 2019). Comprehensive identification of LRR‐RLKs and their resolved phylogenetic relationships will facilitate the further exploration of genes with potentially redundant functions (Nimchuk *et al.*, 2015; Rodriguez‐Leal *et al.*, 2019).

Several factors contribute to incomplete phylogenetic characterization. The primary obstacles are the large size of this family, and that domains found in LRR‐RLKs are also found in many other gene families, resulting in searches that recover over 1000 strong hits per genome (Shiu & Bleecker, 2001a; Lehti‐Shiu & Shiu, 2012). Current phylogenetic methods and approaches are not well suited to resolving gene trees at this scale, so thresholding of results to exclude poor hits must precede phylogenetic inference (Soltis & Soltis, 2003; Lemoine *et al.*, 2018). Typically, the LRR‐RLK family is divided into 15–20 smaller subclades (numbered with Roman numerals), but an ideal thresholding cutoff to isolate subclade members is not always clear (Shiu & Bleecker, 2003; Fischer *et al.*, 2016; Dufayard *et al.*, 2017). Known outgroups, semi‐arbitrary *E*‐values, or reciprocal blasting and clustering have all been used to address this challenge, with variable success (Frickey & Lupas, 2004; Kim *et al.*, 2008; Horiike *et al.*, 2016). We noticed that all current methods preferentially collect full‐length LRR‐RLKs and that there was no record of a systematic attempt to discover a more complete set of structural variants.

To address this issue, we developed a revised approach to discover and phylogenetically characterize all LRR‐RLKs in nine representative embryophyte genomes. Our approach revealed additional genes in the LRR‐RLK superfamily, many of which have uncharacterized structural variation. Some of these new genes had been assigned to other gene families but are actually well‐supported members of the LRR‐RLK family. Using these well‐resolved clades, we used a reduced but representative subset of genes to resolve deep nodes of the LRR‐RLK gene tree and clarify the interclade relationships in this gene superfamily. Although developed for LRR‐RLKs, our technique provides a roadmap for comprehensive gene discovery, and for inferring complete gene trees of large and complex gene families.

## Materials and Methods

### Gene discovery

Primary transcript peptide annotation databases for *Arabidopsis thaliana*, *Amborella trichopoda*, *Brachypodium distachyon*, *Oryza sativa* (rice), *Solanum lycopersicum* (tomato), *Populus trichocarpa* (poplar), *Selaginella moellendorffii* and *Physcomitrella patens* and the longest transcript variant for *Zea mays* (maize) were obtained from Phytozome v.12 and merged into a single peptide database (Goodstein *et al.*, 2012) (Supporting Information Tables S1, S2). Searches for new genes were conducted by collecting previously identified sequences (search priors) and using these to find matches in the peptide database. Original search priors were collected from the results of Dufayard *et al. *(2017) using genes from *A. thaliana*, rice, tomato, and *B. distachyon* in each subclade. Search priors were aligned using Mafft v.7.313 (Katoh & Standley, 2013). From these alignments, overlapping subalignments of *c.* 140 amino acids were extracted manually in geneious v.10.0.8 (Kearse *et al.*, 2012). We found that this length was best for recovering the most true matches. Each subalignment was used to search our peptide genomes using Blast v.2.2.22 and hidden Markov model (HMM) profiles using hmmer v.3.1b2 (Altschul *et al.*, 1990; Eddy, 2011).

### Dynamic discovered gene thresholding

Thresholding search results from smaller subalignments created a new challenge, because some gene regions have very poor conservation (i.e. the LRR N‐terminal cap) whereas others are strongly conserved (i.e. the ATP binding pocket). Thresholding each of these subalignments using the same *E*‐value did not yield consistent results; therefore, we developed a new dynamic thresholding strategy that allowed the collection of genes with closely matching sections without bias against structural rearrangements and, importantly, without lowering thresholding stringency. First, using Blast v.2.2.22, each subalignment's search prior sequences were used to search against the full search prior sequences of the clade to find typical *E*‐values for that particular subalignment (Altschul *et al.*, 1990). Once typical *E*‐values for each subalignment were empirically determined, the full list of search results was subjected to the same search, and the resultant list thresholded according to each search result gene's best hit *E*‐value.

### Iterative phylogenetic inference of subclade gene trees

Thresholded search results for each subalignment were first consolidated into separate LRR results and RLK results. Peptide sequences from all genes in these results were aligned using mafft v.7.313 and viewed in geneious v.10.0.8, where the aligned LRR or RLK domains were extracted manually (Kearse *et al.*, 2012; Katoh & Standley, 2013). This alignment was filtered for homoplastic positions by noisy v.1.5.12 and tested for best substitution model and used to infer a maximum‐likelihood gene tree and 1000 bootstrap replicates using iqtree v.1.6.3 (Dress *et al.*, 2008; Nguyen *et al.*, 2015). The tree was interpreted and visualized using package ggtree v.1.10.0 in R v.3.4.3 (R Core Team, 2017; Yu *et al.*, 2017). The branch length of the outgroup in each tree was set to 1.0 for visual clarity. To search more thoroughly, all genes in the resultant phylogenetic tree with maximum likelihood bootstrap support > 75% as members of the clade were collected and reused as search priors in another round of searching. This iterative process was repeated until the gene family stabilized without any additional genes, generally after two search rounds (Table S3). Final trees were inferred using whole gene alignments generated with Mafft v.7.313 (Dataset S1), filtered for homoplastic positions by noisy v.1.5.12 (Dataset S2), and tested for best substitution model and used to infer a maximum‐likelihood gene tree with partitioned analyses and 1000 bootstrap replicates using iqtree v.1.6.3 (Dataset S3) (Dress *et al.*, 2008; Katoh & Standley, 2013; Nguyen *et al.*, 2015).

### Gene domain calling

Gene domains compiled in the Pfam protein profile HMM database v.31.0 were detected using hmmer v.3.1b2, using the ‘trusted cutoff’ bit score gathering threshold and read into R v.3.5.1 using the package rhmmer v.0.1.0 (Eddy, 2011; Finn *et al.*, 2016; R Core Team, 2017; Arendsee, 2017). The domains found for all transcript variants of each genome were consolidated under the primary variant using the R package dplyr v.0.7.6 (Table S3; Wickham *et al.*, 2019). Results were mapped to trees using tools in the R package ggtree v.1.12.0 (Yu *et al.*, 2017).

### Cryptic domain search

The flanking genomic sequence and coding genes for all putative domain loss genes were collected using a custom R script and bedtools v.2.26 from the nucleotide genome assemblies from which the peptide annotations were derived (Quinlan & Hall, 2010; Goodstein *et al.*, 2012). The sequences were translated in all six reading frames using emboss transeq v.6.6 and scanned for protein domains using Pfam protein profile HMM database v.31.0 and Hmmer v.3.1b2 (Rice *et al.*, 2000; Eddy, 2011; El‐Gebali *et al.*, 2019). All potential cryptic domains were assessed manually for validity and scored as found if the cryptic domain was adjacent to the target gene, in the correct orientation, and not part of a separately annotated full‐length LRR‐RLK (Table S3).

### Expression analysis

Gene expression profiles were collected for poplar from PopGenIE (Sundell *et al.*, 2015), for tomato from the Tomato Expression Atlas (Fernandez‐Pozo *et al.*, 2017), for *A. thaliana* from Thalemine (Krishnakumar *et al.*, 2017), for maize from qTeller (Schnable, 2014), and for rice from the Rice Annotation Project (Kawahara *et al.*, 2013). Correlation of gene pair expression was analyzed using Pearson's product‐moment correlation (Table S4; Best & Roberts, 1975). For generating density plots, the maximum expression in any tissue type was used for each gene and plotted using the R package ggridges v.0.5.1 (Table S4; Wilke, 2017).

### Backbone tree inference

A reduced but representative set of genes from each of our fully resolved LRR‐RLK clade gene trees was used to build a constrained phylogenetic tree of the LRR‐RLK superfamily (Figs S1–S17; Table S5). From these, a constraint tree was constructed from nodes in clade trees with higher than 70% bootstrap support. In most cases, whole clades were constrained, although there were exceptions (Table S5). We selected an outgroup of distant kinase‐containing plant genes (Table S5). These sequences were aligned using Mafft v.7.313 and filtered for homoplastic positions by noisy v.1.5.12 (Dress *et al.*, 2008; Katoh & Standley, 2013). partitionfinder2 v.2.1.1 was used to find appropriate models of protein evolution and the backbone phylogenetic tree was inferred using raxml v.8.2.12 with 1000 fast bootstrapping replicates and thorough maximum likelihood search (Datasets S4–S9; Stamatakis, 2014; Lanfear *et al.*, 2017). These analyses were implemented in cipres (Miller *et al.*, 2015). The tree was interpreted and visualized using packages ape v.5.0 and ggtree v.1.10.0 in R v.3.4.3 (Paradis *et al.*, 2004; R Core Team, 2017; Yu *et al.*, 2017). Transfer bootstrap expectation (TBE) was calculated using Booster using the majority rule tree and bootstrap trees from RAxML (Lemoine *et al.*, 2018).

### Data availability

All supporting information, including the alignments, phylogenetic trees, and HMMs, are available as a Dryad data repository (https://doi.org/10.5061/dryad.jm63xsj6m). Code for analyses and figure generation is available on GitHub (https://github.com/BartlettLab/LRR‐RLK_Evolution).

## Results

### Revised gene discovery method detects typical LRR‐RLK family members and new putative members with structural variation

**Figure 1:**
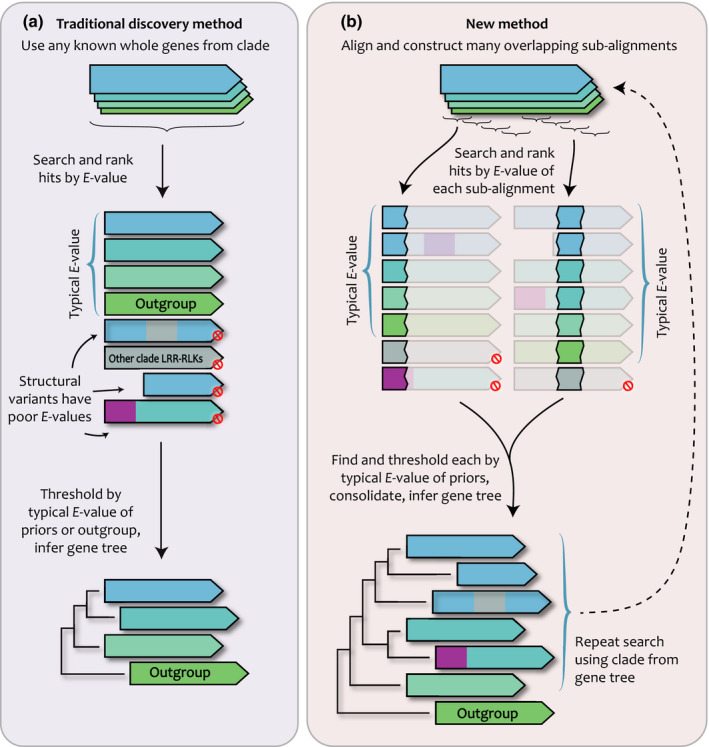
Our gene discovery method was developed for large gene families containing members with structural rearrangements. (a) Traditional gene discovery methods use whole genes as search priors. This penalizes genes with structural rearrangements such as conversions from another clade (gray) or fusions to unrelated domains (purple). Modified genes are ranked below any reasonable threshold and are missed. (b) Our new method utilizes subalignments of known genes as search priors, and ranks hits based on the *E*‐values found, and thresholds at *E*‐values typical for the inputs. Each subalignment search is performed in parallel, and the resultant lists are consolidated and used to construct a gene tree. From the gene tree, all genes in the target clade are again collected and used as priors in another search iteration.

We observed that published gene trees inferred after traditional genome search strategies resulted in bias towards full‐length genes without structural modifications (Fig. 1a). Therefore, to find all LRR‐RLK family members, including structural variants, we developed a new gene search strategy. Our search started with the published genes from Dufayard *et al. *(2017) for each named clade. To avoid search rank penalties for structurally modified genes, we aligned input genes from each clade (search priors) and fragmented these alignments into smaller overlapping subalignments, each of which was used to search for new hits in target genomes (Fig. 1b). We found that subalignment fragment sizes resulting in *c.* 140 amino acids in length were optimal; smaller fragments did not result in more genes collected, and larger fragments missed some. This resulted in an average of four LRR subalignments and three RLK subalignments for each clade. We built HMMs for each subalignment and used these to scan our merged peptide genome database. Hits from these searches were evaluated for *E*‐values typical of the search priors and this *E*‐value threshold was used to exclude weaker hits. Thresholded hits were consolidated across the subalignments and used for phylogenetic inference under the maximum likelihood criterion. This searching and gene tree inference was repeated until no new genes were recovered as well‐supported members of a particular clade (≥ 75% bootstrap support), generally after two rounds (Fig. 1b).

**Figure 2:**
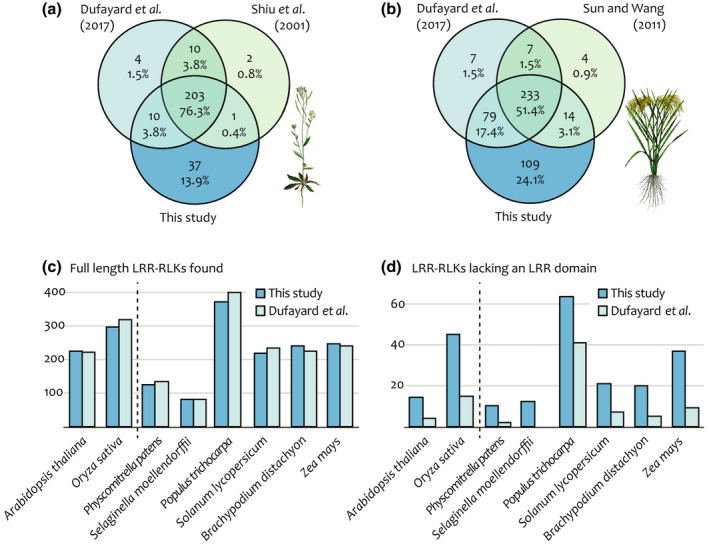
Our gene discovery method revealed new members of the leucine‐rich repeat receptor‐like kinase (LRR‐RLK) family. The number of (a) *Arabidopsis thaliana* and (b) rice genes found here and in other studies. (c) The number of full‐length LRR‐RLKs in each genome is similar to the results of Dufayard *et al.* (2017) and does not explain the difference in number of genes detected. (d) Relative to Dufayard *et al.* (2017), we detected more genes without LRR domains in every taxon.

Relative to other search efforts, we detected nearly all previous LRR‐RLK gene family members and substantially more undescribed ones, including in the high‐quality genomes of *A. thaliana* and rice (Fig. 2a,b; Table S6). To investigate the nature of the additional genes we recovered, we scanned all search results with the Pfam HMM library to detect protein domains (Finn *et al.*, 2016). For each taxon, the number of genes we detected that encode canonical full‐length LRR‐RLKs was similar to the results of other efforts, such as Dufayard *et al. *(2017), suggesting that additional members are not canonical genes (Fig 2c; Tables S3, S7). Dufayard *et al. *(2017) also reported genes in their trees with no LRR domain detected; our results contained many more genes without LRR domains, suggesting that our search method is effectively capturing additional structural variants (Fig. 2d).

To explore these structural variants, we looked for putative domain losses and gains in our domain scan results. We found that genes lacking LRR domains were not the only structural variants; many genes in our trees lack either LRR or RLK domains, or are small fragment genes lacking both LRR and RLK domains, or have additional unrelated domain types (Fig. S18; Tables 1, S3, S7). LRR‐only genes were the most common structural variant, followed by RLK‐only genes (Tables 1, S7).

**Table 1:** Summary of leucine‐rich repeat receptor‐like kinase (LRR‐RLK) structural variant types.

Structural variant	No. genes	Total (%)
All genes	2536	100
Canonical LRR‐RLKs^1^	1887	74.4
LRR‐only genes	271	10.7
RLK‐only genes	252	9.9
Genes potentially from fission	71	2.8
Genes with other domain types^1^	442	17.4
Genes with other domain types^2^	71	2.8

^1^ Including malectin‐like domains in Clades I and VIII‐2.

^2^ Excluding malectin‐like domains in Clades I and VIII‐2.

**Figure 3:**
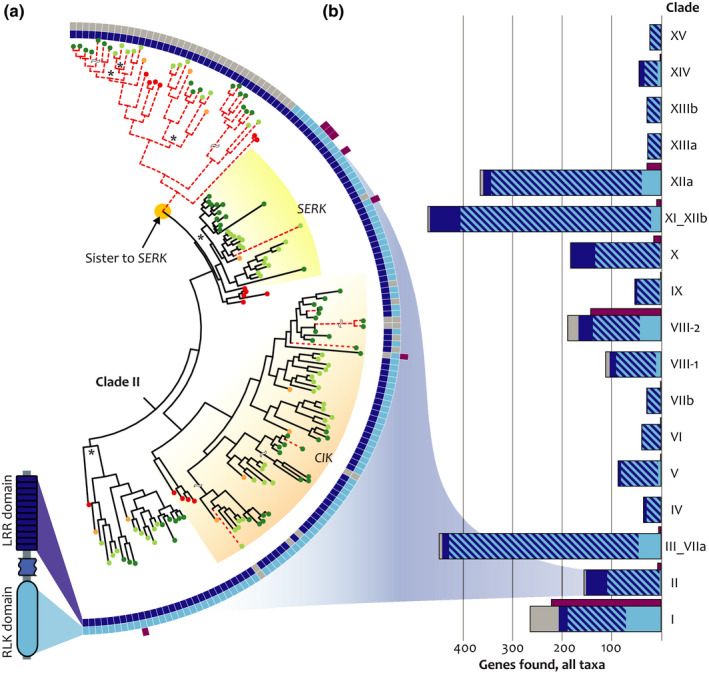
Structural modifications are common in the leucine‐rich repeat receptor‐like kinase (LRR‐RLK) family. (a) Clade II contains many structural variants. Squares on tree perimeter show the detected LRR (dark blue), RLK (light blue), and unrelated (purple) domains. A search using whole genes misses some members (dashed red lines), most notably a large clade of LRR‐only genes sister to the *SOMATIC EMBRYOGENESIS RECEPTOR KINASE (SERK)* clade. All bootstrap support values < 70% marked with asterisk (see Supporting Information Fig. S2 for details). (b) Number of structural variants found in each clade (sum of nine species). Some clades are highly biased towards particular modifications, such as Clades II, X, and XI_XIIb (many LRR‐only genes) and Clades I, III_VIIa, VIII‐2, and XIIa (many RLK‐only genes). Purple bars depict the total number of genes with another domain type found, irrespective of LRR and RLK domain. Clades I and VIII‐2 have ancestral malectin‐like domains that are represented in purple bars.

To check that fragmenting gene alignments was responsible for discovering putative new LRR‐RLKs, we performed another search for Clade II genes using HMMs built from the same input alignment but without fragmentation into subalignments. This whole‐gene search recovered the same set of canonical full‐length LRR‐RLKs but missed most structural variants (Fig. 3a). Therefore, our gene fragmentation search strategy is responsible for the increase in discovery rate of genes with structural variation.

Genes lacking LRR or RLK domains were detected throughout the trees we inferred (Fig. 3a,b; Table S3). Most of these modifications were isolated to single genes in our sampling, but we found some domain losses in expanded clades of conserved genes that predate the divergence of the taxa in our dataset (Figs S2, S3, S6, S12, S16). The most striking domain loss is in Clade II, in a subclade recovered as sister to the well‐characterized *SOMATIC EMBRYOGENESIS RECEPTOR KINASE* (*SERK*) genes (Figs 3a, S2; Meng *et al.*, 2016; Hohmann *et al.*, 2018). These uncharacterized LRR‐only genes have a predicted signal peptide targeting the plasma membrane but no detected transmembrane domain. Genes from all sampled genomes are found in this subclade, indicating that this domain loss is deeply conserved in embryophytes.

### Most LRR‐RLK structural variation cannot be explained by annotation errors

**Figure 4:**
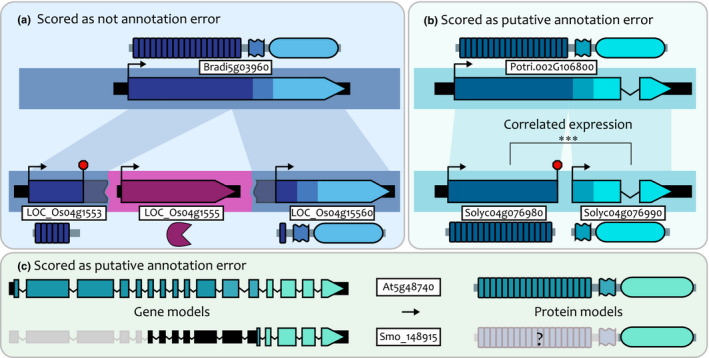
Examples of gene structural evolution and putative annotation errors. (a) An example scored as gene fission. Two rice genes are orthologous to a *Brachypodium distachyon* gene but have been split by an inserted protease gene (purple), with derived start and stop codons now defining the new gene boundaries. High‐scoring alignment regions shown as shaded bars connecting genes. (b) An example scored as annotation error, in which two putatively truncated tomato genes have adjacent arrangement and correlated expression (***, Pearson's *⍴* > 0.7, *P* < 0.001). (c) An example scored as annotation error in a *Selaginella moellendorffii* gene. This is a receptor‐like kinase‐only gene in our gene tree, but has a long 5′ untranslated region (black bars) with conserved sequence and introns with the coding sequence of its full‐length ortholog in *Arabidopsis thaliana*. We scored this class of domain losses as putative annotation errors.

Because our searches were based on peptide annotations, we could not detect unannotated genes, and annotation errors could cause apparent structural variation in translated peptides. For example, misannotation of an LRR domain as a 5′ untranslated region could result in an apparent RLK‐only gene that contained a cryptic LRR domain nearby. To check for these types of annotation errors, we examined nearby sequence of all apparent domain‐loss genes for cryptic coding domains. To do this, we scanned the flanking intergenic nucleotide sequences of all domain‐loss genes using the Pfam protein domain library (Finn *et al.*, 2016). All cryptic domains detected in these scans were investigated manually and scored as possible annotation errors if the domain was adjacent to the target gene, in the correct orientation, and not part of a separately annotated full‐length LRR‐RLK (Table S3). In some cases, cryptic domains were found in such a way that they could not reasonably encode full length LRR‐RLKs (Fig 4a). In other cases, possible annotation errors could not be ruled out (Fig 4b,c).

Our cryptic domain screen revealed that 52 out of 271 (19%) LRR‐only genes had potential unannotated RLK domains, and that 48 out of 252 (19%) RLK‐only genes had potential unannotated LRR domains, but these were not evenly distributed among the nine species tested (Tables 2, S3). We found that the number of potential annotation errors is not proportional to genome size, but instead likely related to genome assembly quality (Table 2). *Arabidopsis thaliana* has the best quality genome assembly, and lowest rate of cryptic domains found, whereas *A. trichopoda* has an underdeveloped genome assembly and the highest rate of possible errors (Table 2). The genomes of rice and poplar are both of average size for this study, and had similar numbers of putative gene truncations, but rice has a very high quality genome and low cryptic domain rate (6%), whereas poplar has a much higher cryptic domain rate (33%) (Tables 2, S3). Despite common annotation errors in some genomes, every genome we surveyed encodes truncated LRR‐RLKs that cannot be explained by annotation errors.

**Table 2:** Rate of possible annotation errors explaining putative gene truncations in each genome.

	Putative gene truncations	No. potential cryptic domains found (%)	Approx. genome size (Mbp)^1^
*Amborella trichopoda*	49	36 (73)	706
*Arabidopsis thaliana*	36	0 (0)	135
*Brachypodium distachyon*	45	2 (4)	272
*Oryza sativa*	114	7 (6)	372
*Physcomitrella patens*	16	1 (6)	472
*Populus trichocarpa*	109	36 (33)	423
*Selaginella moellendorffii*	30	9 (30)	213
*Solanum lycopersicum*	46	15 (33)	835
*Zea mays*	65	7 (11)	2170

^1^ Genome size estimates from Phytozome (Goodstein *et al.*, 2012).

### Most structural variants are expressed at levels typical of validated genes

**Figure 5:**
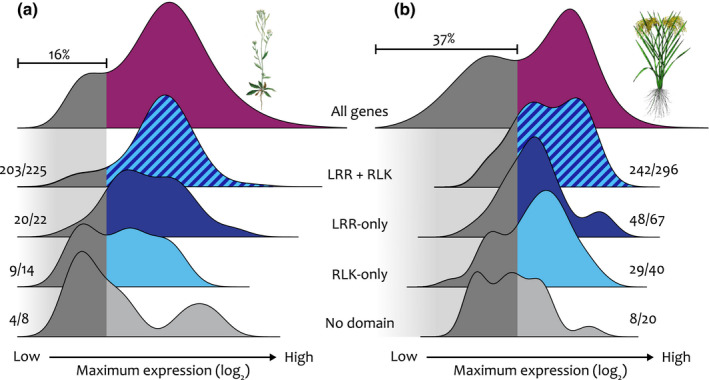
Most structural variants are expressed at similar rates to validated gene models. (a) In *Arabidopsis thaliana*, the maximum expression density curve for all genes has a bimodal distribution, with 16% of all genes having lower maximum expression (dark gray) and the remainder with higher maximum expression (purple). (b) In rice, 37% of all genes have lower maximum expression. Canonical leucine‐rich repeat receptor‐like kinases (LRR‐RLKs) in both species (blue stripes) nearly all have higher maximum expression, as do most LRR‐only LRR‐RLKs (dark blue) and RLK‐only LRR‐RLKs (light blue). LRR‐RLKs without a detected LRR or RLK domain (light gray) are more likely to have lower maximum expression. Numbers to the side of density curves show the fraction of genes in the higher maximum expression range.

The additional LRR‐RLKs we found are annotated gene models, but in order to influence plant traits they must be expressed. In maize, validated gene models tend to be expressed at higher levels, compared with all of the gene models in the genome (Walley *et al.*, 2016; Liang *et al.*, 2019; Schnable, 2019). To test whether the genes we have uncovered are expressed, and whether they have expression levels similar to validated gene models, we condensed the expression profile of all rice and *A. thaliana* genes from diverse RNA‐sequencing datasets into density plots of their maximum expression (purple distributions in Fig. 5; Table S4) (Kawahara *et al.*, 2013; Krishnakumar *et al.*, 2017). As in maize (Walley *et al.*, 2016), we observed bimodal expression distributions for both *A. thaliana* and rice genes (Fig. 5a,b). The expression of most canonical LRR‐RLKs lies towards the higher end of the distribution, as does the expression of most LRR‐only and RLK‐only genes, but most small fragment genes are expressed at lower levels (Fig. 5a,b). Therefore, most LRR‐RLK structural variants, except for the small gene fragments, are expressed at levels similar to validated gene models.

### Some genes classified into other families are modified LRR‐RLKs

Genes with superficial similarities to LRR‐RLKs, such as the leucine‐rich repeat receptor‐like proteins (LRR‐RLPs) are generally treated as separate gene families, despite their similarities to LRR‐RLKs (Fritz‐Laylin *et al.*, 2005; Mondragon‐Palomino & Gaut, 2005; Fan *et al.*, 2018; He *et al.*, 2018; Jamieson *et al.*, 2018). We asked if any genes recovered in our search encoded LRR‐RLPs and found 15 out of 57 (26.3%) *A. thaliana* LRR‐RLPs in our LRR‐RLK trees, many of which have orthologues in other species (Table S3; Fritz‐Laylin *et al.*, 2005; Lv *et al.*, 2016). We also looked for the 14 *A. thaliana* RLK‐only genes from our trees in others’ receptor‐like cytoplasmic kinase searches, but we found none (Table S3; Shiu *et al.*, 2004; Fan *et al.*, 2018). Seven of the RLK‐only genes we found are in Clade I and encode a predicted malectin‐like ectodomain, and are recovered in similar LRR‐RLK searches based on RLK domains (Sun & Wang, 2011; Dufayard *et al.*, 2017; Liu *et al.*, 2017). The remaining seven *A. thaliana* RLK‐only genes are found in other clades and do not encode ectodomains, do not have cryptic LRR‐encoding regions in their adjacent genomic regions, and are not recovered in other searches (Table S3). Therefore, we know very little about RLK‐only genes that originated from LRR‐RLK gene truncations, but they are present in all genomes surveyed.

### Some LRR‐RLKs are the products of gene fusion and fission

**Figure 6:**
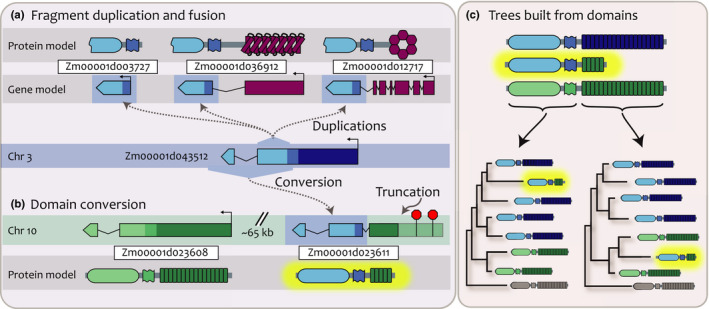
A leucine‐rich repeat receptor‐like kinase (LRR‐RLK) in maize has many structurally modified paralogues. (a) A *c.* 200aa fragment of Zm00001d043512 (blue, Chr 3) is found in three other loci, including a small gene fragment (Zm00001d003727), as a fusion to a pentatricopeptide repeat gene (purple, Zm00001d036912), and as a fusion to a potassium channel gene (purple, Zm00001d012717). (b) A more distantly related LRR‐RLK (green, Zm00001d023608) and its tandem duplicate copy Zm00001d023611 are on Chr 10. The RLK domain of the copy has been converted by Zm00001d043512; it also has a truncation caused by a new start codon, with pseudogenization of the remainder of its LRR domain. (c) Gene trees built using different domains show that the two domains of Zm00001d023611 have different paralogues.

Gene fusion is an important driver in the evolution of multidomain proteins (Pasek *et al.*, 2006; Bailey *et al.*, 2018). Of the genes in our trees, 71 (2.8%) had an unrelated domain type detected in our scans (Tables 1, S3, excluding malectin‐like domains found in Clades I and VIII‐2). Many of these were clustered in hot spots on gene trees, for example a clade of five maize genes in Clade XI_XIIb (Figs 6, S12). Only one of these is a canonical LRR‐RLK, another is a small fragment of the RLK domain, and two are fragments fused to unrelated gene types (Fig. 6a). The fifth is a product of both a conversion and truncation, and therefore cannot be placed into a single correct position on a gene tree (Figs 6b,c, S19; Dataset S10). Other than the small gene fragment, all of these structural variants are expressed at high levels (Table S4). The four structural variants are not shared by any other plant with a sequenced genome, including the close maize relative *Sorghum bicolor*. Only the full‐length variant is present in other published gene trees, illustrating that our method can discover new gene forms.

Gene fission, in which a gene with multiple domains is split into separate genes, might be a major mechanism for generating truncated paralogues (Fig. 4b; Pasek *et al.*, 2006). Gene fission has been characterized in the related NBS‐LRR gene family but not in LRR‐RLKs (Zhong & Cheng, 2016). We found 71 (2.8%) genes in our dataset that are either the product of fission or are annotation errors that split single coding sequences into separate genes (Tables 1, S3). To discriminate between annotation errors and true fission events, we analyzed expression data, reasoning that different expression patterns would indicate true fission events. Only the genomes of tomato, maize, and poplar had both potential fission events and expression data, so these were analyzed for expression correlation and levels relative to genomic background rates (Table S4). In maize, the genes from potentially split annotations were expressed either at high levels or near the threshold for significant expression, and only one pair was annotated as a single gene in some inbred lines (Schnable, 2014; Liang *et al.*, 2019; Monnahan *et al.*, 2019). We found that, overall, *c.* 53% of these pairs had correlated expression patterns (Pearson's *⍴* > 0.7; Table S4), suggesting that annotation errors may explain some apparent gene fissions, but that this type of evolution does drive some structural variation.

We detected no domains of any kind in about 4% of the genes in our results, yet these genes are placed into our gene trees with strong support (Tables 1, S3, S7). These gene fragments are typically short (< 150 aa) and have very high coding sequence identity to their paralogues but very poor sequence identity immediately outside of the annotated coding region (Fig. S20). These could be genome assembly errors, but we found these fragments in all nine genomes examined (Table S3), and many are expressed (Table S4). Therefore, many of these genes are putative LRR‐RLK variants but are typically overlooked in gene searches (Fig. 3a).

### A reduced representation backbone gene tree helps to resolve the deep LRR‐RLK superfamily nodes

**Figure 7:**
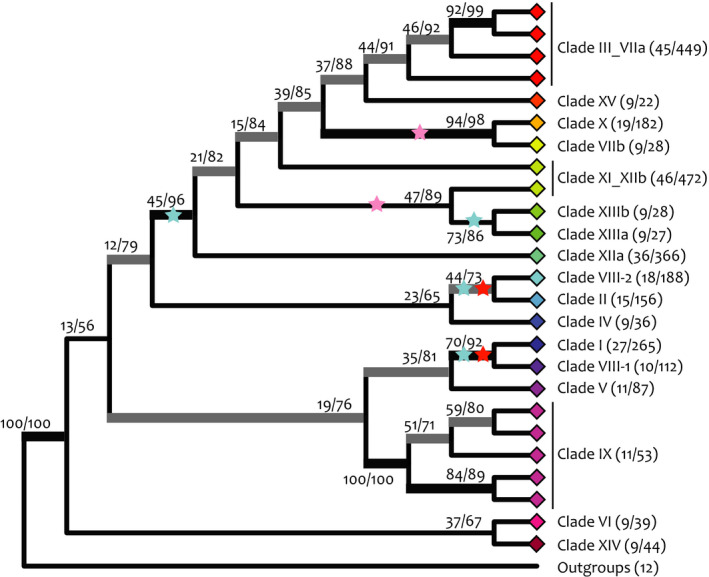
Phylogenetic relationships between leucine‐rich repeat receptor‐like kinase (LRR‐RLK) clades. Representative LRR‐RLKs from well‐supported nodes in the clade‐specific gene tree (> 70% bootstrap support) were constrained in the inference of the backbone gene tree. Felsenstein bootstrap support and transfer bootstrap expectation (TBE) support are shown on branches. Black, thickened branches show > 70% bootstrap support and > 70% TBE support. Gray, thickened branches show > 70% TBE support only. Each diamond represents a collapsed constrained clade. Numbers in parentheses next to clades indicate the number of genes used to infer this tree out of the total number of genes in that clade. Clade relationships confirmed (red stars) and partially confirmed (pink stars) with Shiu & Bleecker (2003). Blue stars, clade relationships confirmed with Liu *et al.* (2017).

We inferred a backbone tree using a reduced set of sequences from each LRR‐RLK superfamily clade. Our resultant backbone resolves the LRR‐RLK genes as monophyletic to the kinase outgroup with 100% bootstrap support, although this may obscure more complex evolutionary relationships with more closely related kinase and LRR‐containing genes (Figs 7, S21; Table S5). Traditional bootstrap support values are low at deep nodes, but bootstrap support may be inappropriate for large numbers of sequences related by ancient nodes (Lemoine *et al.*, 2018). Therefore, we used the TBE metric to assess statistical support for relationships in our backbone tree (Lemoine *et al*, 2018). TBE is based on the number of tips that would need to be removed to recover a given topology and can be interpreted as the proportion of stable tips within a clade. When considering TBE‐supported clades (> 70% TBE: both thick gray and thick black lines in Figs 7, S21) most of the relationships on our backbone gene tree are supported. We compared our tree topology with those of Shiu & Bleecker (2003) and Liu *et al. *(2017). We confirmed four interclade relationships with Liu *et al. *(2017), including a very deep node containing nine clades (blue stars, Fig. 7), and four relationships from Shiu & Bleecker (2003) (red and pink stars, Fig. 7). All but one LRR‐RLK clade (Clade XIIa) includes genes from all nine genomes we searched. These data, together with the backbone tree topology, indicate that the LRR‐RLK clades diverged before the divergence of vascular plants from *P. patens* (Liu *et al.*, 2017). Thus, our gene tree resolves many of the interclade relationships in the LRR‐RLK gene superfamily with more highly supported deep nodes.

## Discussion

Phylogenetic characterization of the LRR‐RLK gene family has been challenged by large family size, copy number variation, limited sequence conservation, and modular domain architecture. To address these challenges, we developed a new method for iterative HMM‐searching and phylogenetic reconstruction of LRR‐RLK subclade trees, and we used these fully resolved gene trees for constructing a curated backbone gene tree of the LRR‐RLKs. We uncovered relationships in the LRR‐RLK superfamily that have gone unnoticed, including putative LRR‐RLK family members with domain truncations and deletions, gene fissions and fusions with unrelated domains, and other structural variation. Uncovering these evolutionary leaps provides insight into the diversification of signaling in plants.

Gene truncations were the most common variant we found (Figs 4, 5; Tables S3, S7). The evolution of a gene encoding a full‐length, multidomain receptor to a single‐domain protein has several interesting implications. Following duplication, paralogues are expected to take one of several different fates, such as neofunctionalization or pseudogenization (Flagel & Wendel, 2009). Truncated paralogues may also retain ancestral function for some time and become partially redundant, especially soon after duplication. Evidence for this comes from a small number of natural events in which truncated LRR‐RLKs retain ancestral function; and synthetic truncations commonly used in biochemical assays can retain specificity for binding partners and ligands (Wang *et al.*, 1998; Ogawa *et al.*, 2008; Song *et al.*, 2014; Meng *et al.*, 2016; Hohmann *et al.*, 2018). If truncated paralogues are both partially redundant and difficult to detect with searches, they may obscure phenotypes while remaining hidden to researchers, frustrating functional studies. For example, the *A. thaliana* Clade VIII‐1 gene *VASCULAR‐RELATED RLK1* (*AtVRLK1*) redundantly regulates secondary cell wall thickening with its full‐length paralogues (Huang *et al.*, 2018). None of these LRR‐RLKs have a single knockout phenotype, and triple knockouts have weak phenotypes, but a dominant negative construct has a strong phenotype, suggesting a functional pathway remains in higher order mutants (Huang *et al.*, 2018). We found an additional *AtVRLK1* gene – At5g49750, truncated to only its LRR domain – that is not present in other gene trees and could retain similar functions to its paralogues. Our method allows for detection of these types of previously invisible paralogues, which may be playing redundant roles in plant signaling.

Another potential fate of truncated paralogues is neofunctionalization. Some truncated variants in our trees are differentially expressed in pathogen screens or even have direct impact on susceptibility to pathogens, suggesting new roles in plant defense signaling (Li *et al.*, 2004; Navarro *et al.*, 2004; Ramonell *et al.*, 2005; Kempema *et al.*, 2007; Ascencio‐Ibáñez *et al.*, 2008; Cartieaux *et al.*, 2008). Another role for neofunctionalized truncated genes may be regulation of full‐length variants via competitive inhibition at the protein level (Seo *et al.*, 2011; Graeff *et al.*, 2016). Truncated proteins called microproteins can heterodimerize with paralogous full‐length proteins to act as interfering regulators (Straub & Wenkel, 2017; Dolde *et al.*, 2018). Some of the truncated and small fragment genes we found resemble microproteins. For example, a previously undetected clade of genes sister to the *SERK*s encode LRR domains and plasma membrane localization signals, but no transmembrane or RLK domains (Fig. 3). Given that the SERKs dimerize with many other proteins as coreceptors to transmit signals, and are competent to do so without their RLK domains, the truncated paralogues may be competing with SERK proteins in oligomeric complexes (Gou *et al.*, 2012; Meng *et al.*, 2016; Zhang *et al.*, 2016; He *et al.*, 2018; Hohmann *et al.*, 2018). Although all characterized microproteins are transcription factors, there is genomic evidence for many classes of microproteins, and a microprotein that interferes with LRR‐RLK function has been successfully engineered; therefore, microprotein function in LRR‐RLKs is plausible (Eguen *et al.*, 2015; Dolde *et al.*, 2018). Because truncated paralogues are intrinsically difficult to detect in large gene families, the technique we outline here can be used to ask if microprotein‐like genes are present in other gene families.

Perhaps the most obvious candidates of truncated and neofunctionalized LRR‐RLKs are the LRR‐RLPs. An early analysis of LRR‐RLK genes in the *A. thaliana* genome found that some LRR‐RLPs clustered with Clades I and II LRR‐RLKs (Shiu & Bleecker, 2003). However, LRR‐RLPs are typically portrayed as a separate monophyletic group (Fritz‐Laylin *et al.*, 2005; Mondragon‐Palomino & Gaut, 2005; Jamieson *et al.*, 2018). Our analysis revealed many more LRR‐RLPs that belong in the LRR‐RLK gene family, beyond the *A. thaliana* LRR‐RLPs in Clades I and II. Some of these are functionally but not phylogenetically characterized; for example, we found that the *RLP2* and *RLP3* genes are truncated paralogues of the full‐length LRR‐RLK gene *PLANT PEPTIDE CONTAINING SULFATED TYROSINE 1 RECEPTOR *(*PSY1R*) in Clade X (100% support, Fig. S11; Mahmood *et al.*, 2014). Many of the *A. thaliana* LRR‐RLPs have orthologues in many species, indicating deep conservation of certain subclades (Table S3). We paid special attention to CLAVATA2 (CLV2), a well‐characterized LRR‐RLP with functional and ligand affinity overlap to the LRR‐RLK CLAVATA1 (Kayes & Clark, 1998; Guo *et al.*, 2010; Je *et al.*, 2018). CLV2 and its orthologues are not recovered in any of our LRR‐RLK searches and are placed as outgroups to any group of LRR‐RLKs in a gene tree, indicating that they are not truncated LRR‐RLKs. However, *RLP2* expressed under the *CLV2* promoter can rescue *clv2* mutants, suggesting convergent structural evolution (Wang *et al.*, 2010). Our analysis demonstrates unambiguously which LRR‐RLPs are truncated variants of LRR‐RLKs, despite the presence of functional and structural similarities.

Of the genes with domain deletions in our dataset, LRR‐only genes are more prevalent and appear to persist longer in genomes once they emerge. We found eight conserved domain losses, six of which resulted in LRR‐only clades and two of which resulted in RLK‐only clades (Figs S1, S2, S3, S4, S12 and S16). The LRR‐only clades have more members per clade and are conserved across deeper evolutionary timescales. We see two explanations for why LRR‐only genes are more likely to be retained: one based on gene structure and one based on protein function. In full‐length LRR‐RLK genes the region encoding the LRR domain is always adjacent to the promoter. Structural deletion of the region encoding the RLK domain is therefore less likely to impact the promoter, whereas a gene with structural deletion of the LRR domain would need to use the now distal promoter or acquire one *de novo*, both substantial obstacles that likely hasten pseudogenization. Another explanation for the differential rate of conservation we observed is that LRR‐only genes may have a greater chance to provide a fitness advantage relative to RLK‐only genes. For example, as pathogens evolve to avoid detection by plant immune receptors, plant receptors are under selection to detect newly evolved signals (Bishop *et al.*, 2000; Anderson *et al.*, 2010; Bailey *et al.*, 2018). But even as LRR domain‐mediated signal detection evolves, the optimum immune response, mediated by RLK domains, may remain the same (Coll *et al.*, 2011; Bashir *et al.*, 2013). LRR‐only domains could evolve quickly to adapt to new signals but still oligomerize with more conserved signal‐transduction machinery. Indeed, based on our alignments, RLK domains are typically more conserved than LRR domains, hinting that LRR‐RLK signaling evolution may occur preferentially through diversifying ligand perception rather than through changes in cellular response.

Gene fusions and fissions have generated many important gene families, including the LRR‐RLKs themselves (Shiu & Bleecker, 2001b; Li *et al.*, 2014). For example, the NBS‐LRR genes, whose LRR domains are distantly related to those found in LRR‐RLKs, are fused to the unrelated NBS domain and are critical components of plant defense (Mondragon‐Palomino & Gaut, 2005; Choi *et al.*, 2016; Bailey *et al.*, 2018). Aside from the deeply conserved malectin‐like domain present in Clades I and VIII‐2, this has not been described within LRR‐RLKs (Feng *et al.*, 2018; Guo *et al.*, 2018). We found that 71 (2.8%) of the genes in our trees contain fusions to other unrelated domain types (Tables 1, S3). The fusion genes we identified are typically only in single taxa and have very high sequence identity to their nearest paralogues. For example, a maize PPR fusion gene has *c.* 96% peptide identity to its nearest paralogue and is not found in any other species (Fig. 6a). Given that we observe these events as recent and infrequent, most fusion genes are probably lost soon after they are generated, though occasionally are deeply conserved, as is the case for Clades I and VIII‐2. Therefore, domain fusions may provide raw materials for selection to act upon in the diversification of plant signaling.

Our analysis shines light on structural variation in the evolution of signaling in plants (Schena & Davis, 1992; Shiu & Bleecker, 2003; Mondragon‐Palomino & Gaut, 2005; van Gisbergen *et al.*, 2018). LRR‐RLKs may be especially prone to genomic restructuring events because of their high copy number, repetitive LRR domains, and high proportion of defense genes (Mondragon‐Palomino & Gaut, 2005; Hofberger *et al.*, 2014; Choi *et al.*, 2016; Hsu *et al.*, 2016). Yet, to our knowledge, this is the first attempt to systematically look for all structural variants in this family. Our work establishes a roadmap for discovering and classifying genes with major structural evolution in any large gene family and highlights the dynamic evolution of plant genomes.

## Author Contribution

MB, JM and JPG conceived and designed the research; JM and JPG performed the research; JM, MB and JPG wrote the manuscript.

## Supporting information

Please note: Wiley Blackwell are not responsible for the content or functionality of any Supporting Information supplied by the authors. Any queries (other than missing material) should be directed to the *New Phytologist* Central Office.

**Dataset S1** Alignments used to infer clade‐specific trees.
**Dataset S2** Alignments used to infer clade‐specific trees after filtering.
**Dataset S3** Newick format clade‐specific tree files.
**Dataset S4** Sequence alignment from backbone tree.
**Dataset S5** Sequence alignment from backbone tree after filtering.
**Dataset S6** Models for backbone tree alignment partitions.
**Dataset S7** Newick format LRR‐RLK constraint tree.
**Dataset S8** Newick format LRR‐RLK backbone best tree.
**Dataset S9** Newick format files for bootstrap replicate trees used in backbone tree construction.
**Dataset S10** Alignments used to construct conversion trees shown in Fig. S19.
**Fig. S1** Clade I gene tree.
**Fig. S2** Clade II gene tree.
**Fig. S3** Clade III_VIIa gene tree.
**Fig. S4** Clade IV gene tree.
**Fig. S5** Clade V gene tree.
**Fig. S6** Clade VI gene tree.
**Fig. S7** Clade VIIb gene tree.
**Fig. S8** Clade VIII‐1 gene tree.
**Fig. S9** Clade VIII‐2 gene tree.
**Fig. S10** Clade IX gene tree.
**Fig. S11** Clade X gene tree.
**Fig. S12** Clade XI_XIIb gene tree.
**Fig. S13** Clade XIIa gene tree.
**Fig. S14** Clade XIIIa gene tree.
**Fig. S15** Clade XIIIb gene tree.
**Fig. S16** Clade XIV gene tree.
**Fig. S17** Clade XV gene tree.
**Fig. S18** Model of structural modifications found.
**Fig. S19** Phylogenetic trees of maize genes in clade XI_XIIb from different alignment domains.
**Fig. S20** Alignment showing sequence identity of a maize gene fragment to its paralog.
**Fig. S21** Backbone tree with gene names.
**Table S1** Genome annotation and assembly versions used in gene searches.
**Table S2** List of maize transcript variants used in gene searches.
**Table S3** All discovered genes, their respective clades, protein domains found in coding annotation, and domains found outside their coding annotation.
**Table S4** Gene expression analyses.
**Table S5** Genes used to construct backbone phylogenetic tree, their clades, and their constraint groups.
**Table S6** Gene family size in each taxon by clade.
**Table S7** Rate of gene structural variation by clade.
