# Supplementary material for: Structural evolution drives diversification of the large LRR‐RLK gene family
Source: New Phytol. 2020 Feb 29;226(5):1492–505. doi: 10.1111/nph.16455 (PMC7318236; doi:10.1111/nph.16455)
Supplement: Supplementary file 1 — Dataset S1 Alignments used to infer clade‐specific trees. Dataset S2 Alignments used to infer clade‐specific trees after filtering. Dataset S3 Newick format clade‐specific tree files. Dataset S4 Sequence alignment from backbone tree. Dataset S5 Sequence alignment from backbone tree after filtering. Dataset S6 Models for backbone tree alignment partitions. Dataset S7 Newick format LRR‐RLK constraint tree. Dataset S8 Newick format LRR‐RLK backbone best tree. Dataset S9 Newick format files for bootstrap replicate trees used in backbone tree construction. Dataset S10 Alignments used to construct conversion trees shown in Fig. S19. Fig. S1 Clade I gene tree. Fig. S2 Clade II gene tree. Fig. S3 Clade III_VIIa gene tree. Fig. S4 Clade IV gene tree. Fig. S5 Clade V gene tree. Fig. S6 Clade VI gene tree. Fig. S7 Clade VIIb gene tree. Fig. S8 Clade VIII‐1 gene tree. Fig. S9 Clade VIII‐2 gene tree. Fig. S10 Clade IX gene tree. Fig. S11 Clade X gene tree. Fig. S12 Clade XI_XIIb gene tree. Fig. S13 Clade XIIa gene tree. Fig. S14 Clade XIIIa gene tree. Fig. S15 Clade XIIIb gene tree. Fig. S16 Clade XIV gene tree. Fig. S17 Clade XV gene tree. Fig. S18 Model of structural modifications found. Fig. S19 Phylogenetic trees of maize genes in clade XI_XIIb from different alignment domains. Fig. S20 Alignment showing sequence identity of a maize gene fragment to its paralog. Fig. S21 Backbone tree with gene names. Table S1 Genome annotation and assembly versions used in gene searches. Table S2 List of maize transcript variants used in gene searches. Table S3 All discovered genes, their respective clades, protein domains found in coding annotation, and domains found outside their coding annotation. Table S4 Gene expression analyses. Table S5 Genes used to construct backbone phylogenetic tree, their clades, and their constraint groups. Table S6 Gene family size in each taxon by clade. Table S7 Rate of gene structural variation by clade. [file NPH-226-1492-s001.zip › Man2019_LRR-RLKs_SupportingInformation/figures/Fig_S17_clade_XV.tree.pdf]

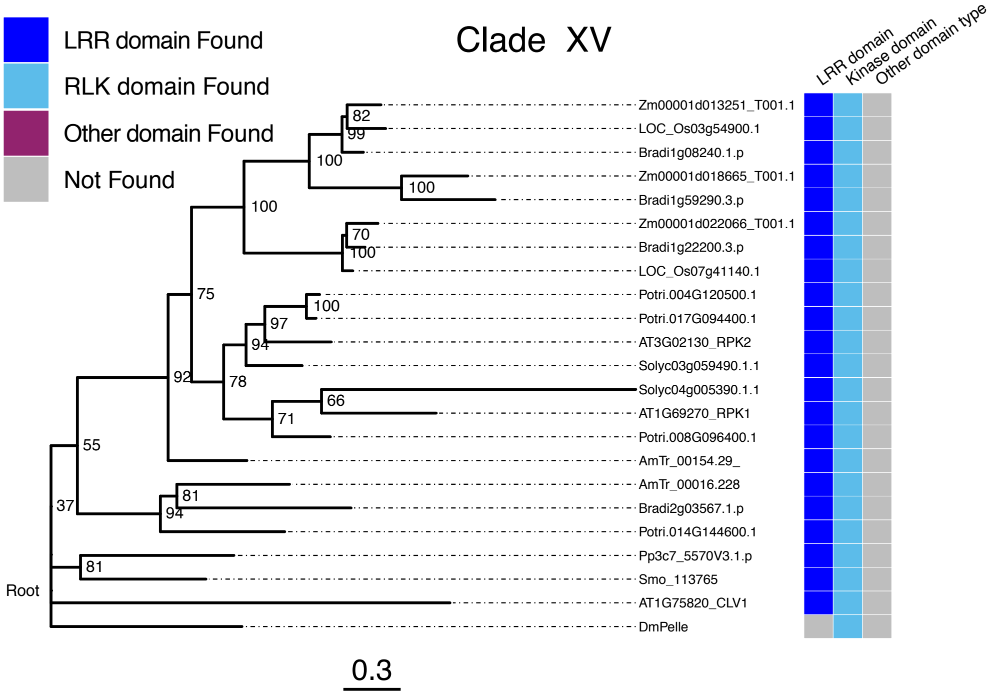

LRR domain Found
RLK domain Found
Other domain Found
Not Found
Clade XV
LRR domain
Kinase domain
Other domain type
Zm00001d013251_T001.1
LOC_Os03g54900.1
Bradi1g08240.1.p
Zm00001d018665_T001.1
Bradi1g59290.3.p
Zm00001d022066_T001.1
Bradi1g22200.3.p
LOC_Os07g41140.1
Potri.004G120500.1
Potri.017G094400.1
AT3G02130_RPK2
Solyc03g059490.1.1
Solyc04g005390.1.1
AT1G69270_RPK1
Potri.008G096400.1
AmTr_00154.29_
AmTr_00016.228
Bradi2g03567.1.p
Potri.014G144600.1
Pp3c7_5570V3.1.p
Smo_113765
AT1G75820_CLV1
DmPelle
Root
0.3
